# Australian Experiences of Out-of-Pocket Costs and Financial Burden Following a Cancer Diagnosis: A Systematic Review

**DOI:** 10.3390/ijerph18052422

**Published:** 2021-03-02

**Authors:** Annie Bygrave, Kate Whittaker, Christine Paul, Elizabeth A. Fradgley, Megan Varlow, Sanchia Aranda

**Affiliations:** 1 Cancer Council Australia, Sydney, NSW 2000, Australia; kate.whittaker@cancer.org.au (K.W.); megan.varlow@cancer.org.au (M.V.); sanchiaa@unimelb.edu.au (S.A.); 2 School of Medicine and Public Health, University of Newcastle, Callaghan, NSW 2308, Australia; Chris.Paul@newcastle.edu.au (C.P.); elizabeth.fradgley@newcastle.edu.au (E.A.F.); 3 Hunter Cancer Research Alliance, Callaghan, NSW 2308, Australia; 4 Department of Nursing, University of Melbourne, Parkville, VIC 3010, Australia

**Keywords:** financial toxicity, cancer patient, systematic review, Australia

## Abstract

(1) Background: This systematic review was conducted to identify cancer patient experiences, and the impact of out-of-pocket costs and financial burden in Australia. (2) Methods: A systematic review, following the Preferring Reporting Items for Systematic Reviews and Meta-Analyses, was conducted. Cumulative Index of Nursing and Allied Health Literature and PubMed were searched. The primary outcome was financial burden among cancer patients and their families in Australia. The secondary outcome was out-of-pocket costs associated with cancer care and treatment within the population sample, and the impact of financial burden. (3) Results: Nineteen studies were included, covering more than 70,000 Australians affected by cancer. Out-of-pocket costs varied by cancer type and ranged from an average of AUD 977 for breast cancer and lymphoedema patients to AUD 11,077 for prostate cancer patients. Younger aged patients (≤65 years), Aboriginal and Torres Strait Islander people, people in rural and/or remote areas, households with low income, those who were unemployed and people with private health insurance were at increased risk of experiencing out-of-pocket costs, financial burden or a combination of both. (4) Conclusions: Australians diagnosed with cancer frequently experience financial burden, and the health and financial consequences are significant. Focusing efforts on the costs of care and options about where to have care within the context of informed decisions about cancer care is necessary.

## 1. Introduction

In Australia, it has been estimated that 146,000 people will be diagnosed with cancer in 2020 [1]. One in two will be diagnosed by their 85th birthday [2]. Consideration of the costs associated with cancer care and treatment, and their true impact on patients is lacking in Australia. The complexities of public and private healthcare services, combined with a patient’s unfamiliarity with the health system can make it difficult for patients to navigate, leading to the issue of unexpected out-of-pocket costs.

In Australia, out-of-pocket costs can range from a few hundred to tens of thousands of dollars, with a landmark patient survey by Consumers Health Forum of Australia reporting that half of cancer patients have out-of-pocket costs of more than AUD 5000 [3]. One in four cancer patients pay more than AUD 10,000 in out-of-pocket costs every 2-years, and one in three pay between AUD 2000 and AUD 5000 [3]. These costs, expected or not, can financially cripple cancer patients and their families, increasing their risk of financial burden, especially when one or more people are unable to work within the household. Increased financial burden due to costs of cancer care and treatment can also be a strong predictor of poor quality of life among cancer survivors [4].

The term “financial burden” is often used interchangeably with financial toxicity, financial distress, financial hardship and economic hardship. However, there is no internationally accepted definition, or one used in Australia. For this review, financial burden refers to the detrimental effects of excess financial strain caused by a cancer diagnosis on the wellbeing and health outcomes of both the patient and their families.

Increasing attention to the extent of financial burden warrants a comprehensive exploration of this complex issue, including understanding where gaps in the literature exist. A systematic review was conducted to identify the experiences of cancer patients, and the impact of out-of-pocket costs and financial burden of cancer care in Australia to inform policy solutions.

## 2. Materials and Methods

### 2.1. Search Strategy

The systematic review was performed following the Preferred Reporting Items for Systematic Reviews and Meta-Analyses (PRISMA) guidelines [5]. Cumulative Index of Nursing and Allied Health Literature (CINAHL) and PubMed were searched for articles published between January 2010 and February 2020, and reference lists of relevant papers were manually examined. An updated search was completed on 1 March 2020.

For all databases, terms were combined from the following themes: (1) cancer; (2) financial toxicity; and (3) Australia. The initial search strategy was developed for PubMed and then adapted for CINAHL. Full details of PubMed search strategy are available in the Supplementary Materials (Table S1).

### 2.2. Inclusion and Exclusion Criteria

Papers that met the following criteria were included: (1) study conducted in Australia; (2) participants affected by cancer, or their families; (3) out-of-pocket costs incurred by population sample and/or financial burden; and (4) papers available in English.

Experimental and non-empirical studies, including editorials, letters, commentaries, and narrative reviews were excluded from selection.

The primary outcome was financial burden among cancer patients and their families in Australia. Out-of-pocket costs associated with cancer care and treatment, and the impact financial burden affecting patients and their families were secondary outcomes.

### 2.3. Screening and Data Extraction

A.B. performed the literature search and undertook the initial screening of the papers based on the title and abstract. A.B. and K.W. independently assessed the full text of the papers, using the eligibility criteria. Disagreements were discussed and resolved through consensus.

The following data were extracted from extracted each study: author(s)—year; participant sample size and demographic factors; study design; diagnosis; data source; outcome measures; time frame; and main findings (Table 1).

A.B. completed the data extraction, with a random sample of studies extracted by K.W. (*n* = 5, 26%). To reduce any bias, a random generator (https://www.random.org/lists/ accessed on 26 February 2021) was used to assign studies. Deferring to K.W. for the remaining papers was considered unnecessary because no discrepancies between reviewers were found.

### 2.4. Data Synthesis and Quality

A.B. and K.W. independently assessed the quality of studies using the National Institute of Health Quality Assessment Tool for Observational Cohort and Cross-Sectional Studies [6]. The tool asks 14 questions relating to the risk of bias in studies. The questions prompt the reviewers to focus on concepts key to critically appraise the internal validity of a study and identify where limitations may exist, as opposed to ranking the overall quality. Reviewers can respond to each question with either a “yes”, “no” or “other”. No studies in the review were excluded based on quality.

A narrative synthesis was completed, which was guided by Carrera and Zafar’s model of Financial toxicity, its attributes and impact and previous work in understanding the individual factors influencing financial burden [7]. Themes were categorised into the following three domains: (1) out-of-pocket costs of cancer care and treatment (direct and indirect); (2) risk factors related to out-of-pocket costs and/or financial burden; and (3) health outcomes associated with financial burden.

**Table 1:** Selected characteristics from included studies.

Author(s)-Year	Participant Sample Characteristics	Study Design	Data Source	Outcome Measures	Out-of-Pocket Costs (Direct and/or Indirect)	Key Findings
Boyages, 2017 [8]	Total of 361 women diagnosed with breast cancer (BC), or BC and lymphoedema (LE) were recruited by Breast Cancer Network Australia and Australasian Lymphology Association.	Cross-sectional online survey	Self-reported	Study-specific survey items Lymphoedema severity scale	Mean OOP cost of LE was AUD 977 (s.d. AUD 111; range AUD 0–AUD 12,000) Average cost of garments per year increased with lymphoedema severity, from AUD 207 for subclinical severity and AUD 1400 for severe severity.	**Financial burden** Coping mechanisms BC and BC+LE pts reported spending less on social activities (24% vs. 24%, respectively) and holidays (21% vs. 19%, respectively). Household income Among the patients, 39% of BC and 34% of BC+LE pts reported reduction in income. **Impact of financial burden** BC+LE pts indicated that their BC diagnosis had significantly affected them financially compared with BC pts only (*p* < 0.020).
Callander et al., 2019 [9]	Total of 25,553 adult pts diagnosed with cancer in Queensland between 1 July 2011 and 30 June 2012 were recorded by the Queensland Cancer Registry.	Cohort	Medical Benefits Scheme, Pharmaceutical Benefits Scheme	CancerCostMod	Indigenous vs. non-Indigenous **0–12 months**: AUD 401 vs. AUD 1074 **13–24 months**: AUD 200 vs. AUD 484 **25–36 months**: AUD 181 vs. AUD 441	**Impact of OOP cost** Indigenous status Indigenous status was a significant predictor of OOP expenditure (*p* < 0.001), irrespective of demographic and social characteristics being adjusted for. Indigenous pts with cancer on average accessed 236 services per person. If Indigenous pts had the same rate of service use as non-Indigenous pts, this would increase to 309 services per person. Socioeconomic status Pts in Q4 and Q5 paid significantly more in OOP expenditure than pts in Q1.
Callander et al., 2019 [10]	Total of 25,553 adult pts diagnosed with cancer in Queensland between 1 July 2011 and 30 June 2012 were recorded by the Queensland Cancer Registry.	Cohort	Medical Benefits Scheme, Pharmaceutical Benefits Scheme	CancerCostMod	Indigenous vs. non-Indigenous **0–6 months:** AUD 269 vs. AUD 732 **7–12 months**: AUD 110 vs. AUD 359	**Impact of OOP cost** Indigenous status After adjusting for confounding factors, Indigenous Australians were 61% less likely to accrue patient co-payments 0–6 months post-diagnosis. They were also 63% less likely to accrue patient co-payments at 7–12 months post-diagnosis.
Dussel et al., 2011 [11]	Total of 89 bereaved Australian parents whose child was cared for at Royal Children’s Hospital, Melbourne between 1996 and 2004.	Cross-sectional	Self-reported	Study-specific survey items Degree of financial hardship National Median Equivalised Income (NMEI)	NR	**Financial burden** Coping mechanisms Fundraising (33%) followed by reduction in consumption (25%) and borrowing money (22%) were coping mechanisms. Employment status Among the Australian families, 88% reported substantial work disruptions, with 49% quitting their job. Household income More than one-third of Australians families lost ≥ 40% of their income, with 22% falling below the poverty line. Pts from the lowest income categories experienced the greatest income loss. **Impact of financial burden** Among the Australian families, 39% reported overall financial hardship due to their child’s illness.
Gordon et al., 2019 [12]	Total of 289 men diagnosed with prostate cancer were recruited from the Prostate Cancer Foundation of Australia.	Cross-sectional, online survey	Self-reported	Study-specific survey items	Median OOP for cancer treatment was AUD 8000 (IQR AUD 14,000)	**Financial burden** Coping mechanisms Drawing on savings (38%), selling assets (8%) and increasing credit card limits (22%) were coping mechanisms used to pay for treatment. Employment status Among the men, 39% reported that they were in the workforce at the time of the diagnosis, and nearly 25% stated they chose an earlier retirement age and had stopped work due to their diagnosis. On average, those who had retired early had retired 4–5 years earlier than planned. **Impact of financial burden** Among the men, 22% found the cost of treating their prostate cancer caused a “great deal” of distress.
Gordon et al., 2017 [13]	Total of 542 colorectal cancer survivors were recruited through the Queensland Cancer Registry between January 2010 and September 2011.	Cohort	Self-reported	Household Income and Labour Dynamics in Australia (HILDA) survey degree of financial hardship	NR	**Financial burden** Employment status Cancer survivors were more likely to report not being financially comfortable if they had ceased or decreased employment, compared to those who maintained or increased employment participation (OR 1.66, 95% CI 1.12–2.44; *p* < 0.05). Higher proportion of workers reported financial strain at 6 months compared to 12 months (15% vs. 7%, respectively; *p* = 0.003).
Gordon et al., 2017 [14]	Total of 5673 pts diagnosed with at least one histopathological keratinocyte cancer or melanoma were recruited from the Queensland electoral roll between 2010 and 2011.	Cohort	Medical Benefits Scheme, Pharmaceutical Benefits Scheme	QSkin Sun and Health study	**Melanoma:** Mean OOP costs -1 melanoma: AUD 559 (s.d. AUD 549) - ≥1 melanoma: AUD 1151 (s.d. AUD 779) **Keratinocyte cancers:** Mean OOP costs -low frequency: AUD 407 (s.d. AUD 598) -high frequency: AUD 1520 (s.d. AUD 1698) **Skin lesions:** Avg. costs ranging from AUD 193–AUD 377	NR
Gordon et al., 2018 [15]	Total of 840 pts diagnosed with either melanoma, prostate, breast, colorectal or lung cancer from the QSkin Sun and Health Study were recruited, at random, from the Queensland electoral roll between 2010 and 2011.	Cohort	Medicare, Queensland Cancer Registry	QSkin Sun and Health study	**Median OOP costs:** AUD 4192 breast cancer, AUD 3175 prostate cancer, AUD 1078 lung cancer. Therapeutic procedures (median: AUD 2062) were largest OOP expenses for pts, followed by professional attendances (AUD 546) and PBS medicines (AUD 428).	NR
Gordon et al., 2020 [16]	Total of 204 pts diagnosed with neuroendocrine tumours were recruited from hospital clinics in Queensland, Victoria and New South Wales, and the Unicorn Foundation.	Cross-sectional, survey	Self-reported	Study-specific survey items EuroQol 5-dimension 5-level (EQ-5D-5L), Comprehensive Financial Toxicity (COST) tools	Mean OOP costs were AUD 1698 (s.d. AUD 2132). Mean OOP costs for medical tests was AUD 376 (s.d. AUD 722), travel-related expenses AUD 289 (s.d. AUD 559) and specialists visits AUD 225 (s.d. AUD 342).	**Financial burden** Employment status One-third of current workers reported that cancer had prevented them from securing employment, and another third reported a decrease in their work hours. Among the pts, 17% said colleagues treated them differently, 16% had not told their employers or work colleagues about their cancer, and 7% were overlooked for promotion. One-third of current workers said they would retire early due to their cancer. Place of residence Among the rural pts, 30% reported travel and accommodation as their largest expense, compared to urban pts (13%). Private health insurance More than two-thirds of pts had private health insurance and of those, 58% stated that insurance did not cover expected expenses. Pts with private health insurance paid more OOP costs than those without insurance for medical tests (18% vs. 7%, respectively) and specialist visits (11% vs. 5%). Treatment Among the pts, 31% reported that cost was a consideration in choosing their cancer treatment course, and 8% did not proceed with treatment due to cost. Among the pts, 60% purchased alternative therapies due to high cost of recommended treatment. **Impact of financial burden** Financial assistance One in five needed financial advice after their cancer diagnosis. Quality of life Overall mean health-related quality of life score for the EQ-5D-5L was 0.65 (s.d. 0.23). Poorer quality-of-life scores were significantly associated with a poorer financial toxicity score (mean 0.53, 95% CI 0.45–0.61; *p* = 0.01), two or more co-morbidities (mean 0.59, 95% CI 0.53–0.66; *p* = 0.02), younger age (mean 0.61, 95% CI 0.55–0.60; *p* = 0.02), not working due to cancer (mean 0.56, 95% CI 0.47–0.65; *p* = 0.03), and nausea/diarrhoea (mean 0.63, 95% CI 0.60–0.67; *p* = 0.01).
Hall et al., 2016 [17]	Total of 4299 haematological cancer survivors were recruited from 5 Australian state population-based cancer registries.	Cross-sectional, survey	Self-reported	Depression Anxiety and Stress Scale 21-item.	NR	**Impact of financial burden** Quality of life Survivors who had less income due to their cancer (OR 1.81, 95% CI 1.10–2.99; *p* = 0.004) and did not have time off work (OR 1.76, 95% CI 1.02–3.02; *p* = 0.012) had higher odds of reporting above normal levels of anxiety compared to their counterparts. Survivors who reported having to use their savings due to cancer diagnosis (OR 1.81, 95% CI 1.07–3.05; *p* = 0.006) or had difficulty paying their bills (OR 1.94, 95% CI 1.03–3.67; *p* = 0.012) had greater odds of experiencing above normal levels of depression. Survivors aged between 50 and 59 years at diagnosis (OR 2.69, 95% CI 1.10–6.56; *p* = 0.008) reported difficulties in paying their bills due to cancer (OR 1.94, 95% CI 1.03–3.67; *p* = 0.012) and having used up their savings due to cancer (OR 1.81, 95% CI 1.07–3.05; *p* = 0.006) had higher odds of reporting above normal levels of stress.
Hall et al., 2015 [18]	Total of 715 haematological cancer survivors were recruited from 4 Australian state population-based cancer registries.	Cross-sectional, survey	Self-reported	Survivors Unmet Needs Survey	NR	**Financial burden** Coping mechanisms Survivors having trouble meeting day-to-day expenses due to their cancer and treatment had higher odds of reporting a “high/very high” unmet need in relation to “dealing with feeling worried” than those who did not (OR 3.1, 95% CI 1.47–6.47; *p* = 0.003). Survivors who reported using up their savings due to cancer and treatment had higher odds of reporting a high level of need for “dealing with feeling tired” (OR 3.0, 95% CI 1.71–5.14; *p* < 0.001) and “coping with having a bad memory or lack of focus” compared to those survivors who did not (OR 2.3, 95% CI 1.07–5.02; *p* = 0.04).
McGrath, 2016 [19]	Total of 45 haematological cancer pts from regional, rural and remote areas of Queensland.	Cross-sectional, phone interviews, qualitative	Self-reported	Study-specific survey items	NR	Place of residence Food was described as a significant cost associated with travel and accommodation for specialist care. Parking at metropolitan hospitals was a significant expense. Many pts described it as being “trapped” without options, other than to pay the high cost of parking for both pts and their carers. Private health insurance For some pts with private health insurance being treated in the private hospital system, the “gap payments” were a problem. Treatment Follow-up treatment drugs after discharge from hospital, including the drugs for symptom relief, the immunosuppressant drugs, the steroids, and maintenance drugs were a significant expense.
McNeil 2018, [20]	Total of 196 adolescents and young adults aged 15–25 years receiving cancer care across Australia between September 2010 and December 2012.	Cohort survey	Self-reported	Study-specific survey items Psychosocial Assessment Tool, Likert scale	NR	**Financial burden** Employment status Among the AYAs, 45% reported they had been able to “get back on track” with work plans, 30% were back on track to some extent and 15% were not able to get back on track. **Impact of financial burden** Financial assistance Among the AYAs, 60% reported it was important for them to receive income support during treatment and 48% reported it was important after treatment. Of those AYAs who needed income support during treatment, 77% reported needing income support after treatment. The need for income support for AYAs during treatment was significantly associated with older age at diagnosis (OR 2.22, 95% CI 1.23–4.01, *p* < 0.01) and being unemployed (OR 3.29, 95% CI 1.28–8.45, *p* = 0.01). AYAs who indicated they did not need government income support reported financial assistance from other sources, including pre-existing employment structures, income protection, parents, and personal savings.
Newton et al., 2018 [21]	Total of 400 pts diagnosed with breast, lung, colorectal or prostate cancer who resided in rural regions of Western Australia (Great Southern Goldfields, South West and Midwest) were recruited through the Western Australia Cancer Registry.	Cross-sectional	Self-reported	Modified version of Paul et al.’s questionnaire Catastrophic spending defined as at least 10% of household income spent on health.	Median OOP cost of AUD 2179 (95% CI AUD 1873–AUD 2518)	**Financial burden** Employment status Among the pts, 19% reported a change in employment circumstances post-diagnosis. Household income One in ten pts experienced catastrophic spending on healthcare, with 7% reporting on OOP costs that equated to 10%–20% of their total household income, 4% reporting 20%–40% and 1% reporting more than 40%.
Paul et al., 2016 [22]	Total of 255 oncology pts were recruited from outpatient clinics at two large hospitals in Australia.	Cross-sectional, questionnaire	Self-reported	Study-specific survey items	NR	**Financial burden** Employment status Among the pts, 67% indicated a change in employment with permanent employment changes. The most frequently reported changes were reduced hours (23.1%), retirement (20.2%) and resigning or being unemployed (16.4%). Household income Among the pts, 63% reported reduced household income since their diagnosis, with a mean reduction in fortnightly income of AUD 752.2 (s.d. AUD 583.60). Private health insurance After adjusting for employment status and age, pts with private health insurance had higher odds of reporting financial factors to influence treatment decision making (OR 2.51, 95% CI 1.27–4.98; *p* < 0.05). Pts with private health insurance had significantly higher odds of reporting that financial factors had influenced their treatment decision making (OR 2.51, 95% CI 1.27–4.98; *p* < 0.05), even after adjusting for employment and age. **Impact of financial burden** Financial assistance Among the pts, 74% reported that they did not access financial assistance, with more than a third (37%) of those being unaware that financial assistance was available. Of those who did receive assistance, government benefits were the most-nominated form of financial assistance (16.7%); 4.9% reported using travel assistance schemes, 5.7% financial support from a Cancer Council, 2% assistance from another cancer organisation, and 0.8% using Cancer Assist. Treatment Travel (15%), loss of income (14%) and treatment cost (11%) were commonly cited factors influencing treatment decision-making.
Paul et al., 2016 [23]	Total of 255 oncology outpatients attending treatment or appointments in Australia were recruited between January and July 2014.	Cross-sectional, questionnaire	Self-reported	Study-specific survey items	NR	**Financial burden** Household income Pts reporting reduced income after being diagnosed with cancer had higher odds of reporting a heavy or extreme financial burden associated with prescribed medicines for cancer (OR 3.73, 95% CI 1.1–12.1, *p* = 0.289). Treatment Among the pts, 63% reported some level of financial burden associated with obtaining prescribed medicines. Of those, 34% of pts reported moderate or heavy financial burden, and 11.8% reported using alternatives to prescribed medicines, such as over-the-counter, medicines from home or medicines from someone else.
Rowell et al., 2016 [24]	Total of 4000 pts diagnosed basal or squamous cell skin cancer were recruited between June 2011 and 2012.	Cohort	Medical Benefits Scheme, Pharmaceutical Benefits Scheme	QSkin Sun and Health study	Avg. cost was AUD 306, of which the public subsidy was AUD 241 and the co-payment was AUD 65.	NR
Slavova-Azmanova et al., 2019 [25]	Total of 40 cancer pts were recruited from the population sample of out-of-pocket expenses study in outer metropolitan and rural areas of Western Australia.	Cohort, phone interviews, qualitative	Self-reported	Study-specific survey items	NR	**Financial burden** Private health insurance Pts with private health insurance expressed disappointment with providers who did not make the option of receiving care as a public patient. Pts treated in the private sector acknowledged that cost of treatment had never been discussed and expressed disappointment with the lack of price transparency and cost-related discussions. Treatment Quality of communication regarding treatment options and side effects of treatment was variable and sub-optimal. **Impact of financial burden** Financial assistance Lack of awareness of services and costs prevented pts from accessing financial assistance, leading to treatment non-adherence and unnecessary stress to pts and their families.
Zucca et al., 2011 [26]	Total of 1410 pts diagnosed with one of the eight most incident cancers in Australia were recruited from New South Wales and Victorian cancer registries.	Cohort	Self-reported	Study-specific survey items	NR	**Financial burden** Place of residence Outer regional/remote pts had the greatest travel burden during the first 12 months after diagnosis. Among the pts, 61% travelled at least 2 h one way to receive treatment, and 49% lived away from home to receive treatment. Strongest associates of travel burden were: - Living in inner regional (OR 18.9, 95% CI 8.41–42.52; *p* < 0.001); - Living in outer regional/remote (OR 135.6, 95% CI 56.96–323.05; *p* < 0.001); - Having received surgery (OR 6.7, 95% CI 2.67–16.95; *p* < 0.001); - Having received radiotherapy (OR 3.6, 95% CI 1.78–7.41; *p* < 0.001). Treatment Between 6 and 12 months after diagnosis, 2% of pts had declined cancer treatment because of the time it took to get to treatment. Even after adjusting for confounding factors, pts who travelled more than 2 h or lived away from treatment reported significantly greater financial difficulties (38% and 40%, respectively) than those who did not (12% and 14%, respectively).

AYAs, adolescents and young adults; BC, breast cancer; CI, confidence intervals; IQR, interquartile range; LE, lymphoedema; NR, not reported; OR, odds ratio; OOP, out-of-pocket; pts, patients; Q1, quintile 1; Q4, quintile 4; Q5, quintile 5.

## 3. Results

### 3.1. Study Selection

**Figure 1:**
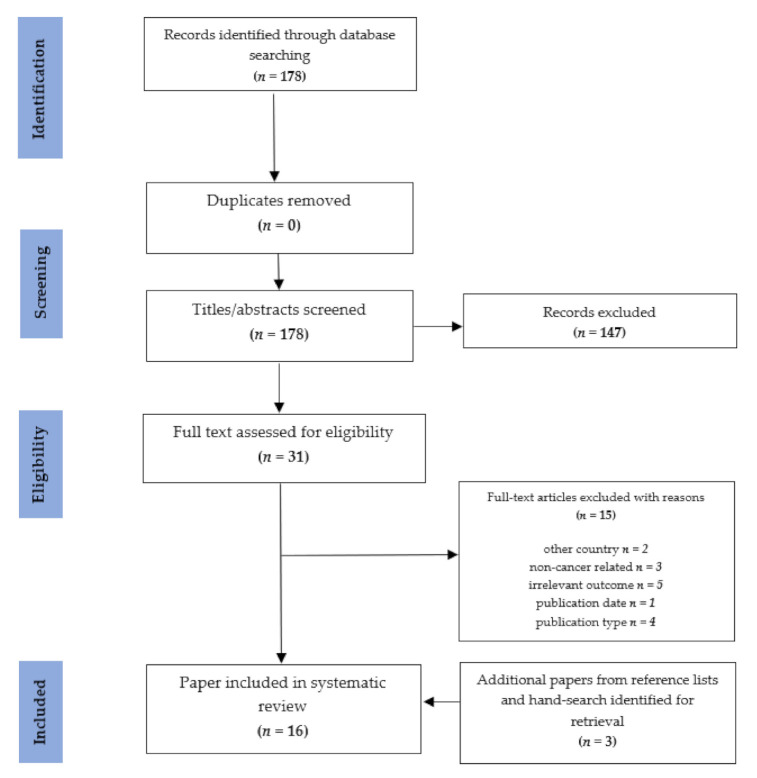
Preferred Reporting Items for Systematic Reviews and Meta-Analyses of the study selection process.

A flowchart of the study selection process is presented in Figure 1. A total of 178 records were retrieved with thirty-one full-text papers assessed; of these, 16 were eligible. Three additional papers were identified in the hand-search of reference lists, resulting in 19 papers included in the systematic review.

The main reasons for exclusion were inappropriate study type, irrelevant topic for the purpose of this review and the cost incurred by a source other than patient (e.g., government or insurer).

### 3.2. Study and Participant Characteristics

Ten studies were conducted Australia-wide, six in Queensland, two in Western Australia and one in Victoria. Cohort and cross-sectional study designs were utilised.

The sample sizes for the included studies ranged between 40 and 25,553 participants (see Table 1). A variety of cancer types were studied, including breast, colorectal, haematological and prostate cancer, keratinocyte, and neuroendocrine tumours.

### 3.3. Financial Burden and Out-of-Pocket Cost Measures

Fourteen studies used self-reported measures, including study-specific survey items and validated measures such as the Comprehensive Financial Toxicity Tool, EuroQoL 5-dimension 5-level, Depression, Anxiety and Stress Scale 21-item. Other studies used CancerCostMod, a model of health service use, healthcare expenditure and patient co-payments for people diagnosed with cancer in Australia [27], and the Medicare Benefits Scheme and Pharmaceutical Benefits Schedule administrative data sets.

### 3.4. Quality of Studies

The quality assessment of the studies is illustrated in Table 2. Generally, studies had clearly stated objectives and specified population samples. Multivariable analyses were also utilised, where possible, to address confounding. Three papers were assessed as low risk of bias, which translated to good quality [9,10,26]. Common areas of weakness across several studies were low response rates (≤50%) and poorly defined outcomes with no validated tools, such as CancerCostMod. Most were cross-sectional studies and measured diverse outcomes, which hampered the comparability of studies.

### 3.5. Out-of-Pocket Costs

Eleven studies reported on out-of-pocket costs, which included direct costs for medical treatment and indirect costs.

#### 3.5.1. Direct Costs

The average (mean or median) total out-of-pocket costs varied from AUD 977 (s.d. AUD 111) for breast cancer and lymphoedema to AUD 11,077 for patients with prostate cancer [8,12].

Another study using cancer patients from the QSkin Sun and Health study found that those who had multiple skin cancers had out-of-pocket costs, ranging from AUD 193 to AUD 377 over 3 years [14]. It was also reported that patients with ≥2 melanomas, or >1 keratinocyte were less likely to not have any out-of-pocket costs than patients who were only treated for one, respectively (53% vs. 41%, *p* = 0.176) (42% vs. 60%; *p* < 0.001) [14].

#### 3.5.2. Indirect Costs

The most commonly reported indirect costs were travel-related expenses (food, fuel, parking) and over-the-counter medicines, but these differed across studies [16,19,20,21,23]. A study of patients with neuroendocrine tumours found that the mean cost for travel-related expenses was AUD 289 (s.d. AUD 559), accounting for 13% of patients total out-of-pocket costs [16]. In the same cohort, 30% of rural patients reported travel and accommodation costs as the largest expense, compared to 13% of urban patients [16]. A qualitative analysis of haematological cancer participants in regional, rural and remote areas of Queensland also identified that parking at metropolitan hospitals was a frequent and substantial financial cost, with some stating that they felt “trapped” in paying the high cost as other options were limited [19].

A cross-sectional survey of women who had lymphoedema as a side effect of breast cancer showed that compression garments accounted for 40% of their out-of-pocket costs [8]. The average cost of compression garments self-reported by participants per year increased with lymphoedema severity, from AUD 98 for subclinical severity and AUD 1000 for severe severity (*p* < 0.001) [8].

### 3.6. Risk Factors Related to Out-of-Pocket Costs or Financial Burden

A total of fourteen studies evaluated the risk factors for financial burden.

#### 3.6.1. Age

Younger age (≤65 years) was associated with worse financial burden, with increasing age associated with decreasing financial burden [17,20]. A study of adolescents and young adults (AYAs) identified that 20–25 years old with cancer reported an increased likelihood of financial issues, compared to 15–19-year-olds (OR 1.98, 95% CI 1.06–3.67; *p* = 0.31) [20]. Regression analyses demonstrated that this may be because younger AYAs were living with their family, which reduced the likelihood of having financial issues (OR 0.5, 95% CI 0.25–0.98; *p* = 0.044) [20].

#### 3.6.2. Indigenous Status

CancerCostMod data demonstrated that Aboriginal and Torres Strait Islander people have significantly less out-of-pocket costs for each 12-month period post-cancer diagnosis compared to non-Indigenous Australians [9,10]. Out-of-pocket costs in the first year after diagnosis were AUD 693 lower for Aboriginal and Torres Strait Islander people [9,10]. They also spend less than non-Indigenous Australians on Medicare services, including pathology tests (≤79%), specialist services (≤75%) and diagnostic imaging (≤74%) [10].

#### 3.6.3. Place of Residence

Cancer patients living in rural or remote areas of Australia were more likely to experience increased financial burden than those in metropolitan areas [19,25,26]. A study of cancer patients in New South Wales and Victoria found that those who travelled more than 2 h or lived away for treatment had greater financial difficulties (38% and 40%, respectively) than those who did not, (12% and 14%) even after adjusting for covariates [26].

#### 3.6.4. Household Income

Lower household income was associated with increased odds of financial burden [8,11,21,22]. Nearly two-thirds of adult cancer patients reported less household income following their diagnosis, with a fortnightly mean reduction of AUD 752.20 [22].

One in ten rural Western Australians diagnosed with breast, colorectal, lung or prostate cancer experienced catastrophic spending on healthcare, with 7% reporting out-of-pocket costs that equated to 10–20% of their total household income [21]. Nearly one-third of Australian families who had a child die from cancer fell below the poverty line due to loss of income [11]. Families from the lowest income category reported the greatest proportion of income loss [11].

#### 3.6.5. Employment Status

Unemployment or job change was an independent risk factor for worse financial burden [11,12,13,21,22]. Nearly three-quarters of middle-aged colorectal cancer patients who had ceased or decreased employment following their diagnoses were not financially comfortable, compared to those who maintained or increased employment participation [12,13].

#### 3.6.6. Private Health Insurance Coverage

Private health insurance was associated with increased odds of higher direct out-of-pocket costs [12,16,19,21,22]. Irrespective of time since diagnosis, prostate cancer patients with private health insurance reported double the out-of-pocket costs than those without private health insurance (AUD 10,052 and AUD 5103, respectively) [12].

### 3.7. Health Outcomes Associated with Financial Burden

The association of financial burden with health behaviours was addressed in four studies, and quality of life in another two studies. Financial burden and psychological distress were associated with nonadherence to cancer-specific treatment which included delaying, modifying, forgoing or not completing recommended treatment.

The overall cost of cancer-specific treatment was associated with financial burden [16,22,23,26], with 8% of neuroendocrine patients reported to forgo treatment completely due to cost [16]. Another study found that 28% of cancer patients reported moderate or heavy financial burden, and of those, 12% used alternatives to prescribed medicines, such as over-the-counter medicines due to cost [23]. Furthermore, cancer patients with private health insurance were more than two times as likely to report that financial factors influenced treatment-decision making, even after adjusting for employment and age [22].

In two studies on psychological distress, evidence suggested that increased anxiety, stress and worry due to financial burden was associated with decreased quality of life [16,17]. Twenty-five percent of haematological survivors reported above normal levels of anxiety and stress, and nearly 20% reported above normal levels of stress [17]. Haematological survivors who had experienced financial burden due to their cancer had higher odds of reporting multiple domains of psychological distress, compared to other cancer survivors [17].

### 3.8. Financial Burden Coping Strategies

Six studies reported the adoption of coping behaviours, such as modifying lifestyles or altering long-term financial plans to pay for cancer treatment. Patients affected by cancer drew on savings, sold assets and increased credit card limits to pay for treatment [11,12,13,17,18]. One-third of Australian families who had a child die from cancer commonly reported fundraising to cover the costs of living and deal with financial distress [11].

A study of women with breast cancer and lymphoedema found that financial burden was associated with spending less on social activities and holidays [10].

## 4. Discussion

This systematic review, capturing the experiences of over 70,000 Australians, found that out-of-pocket costs and financial burden associated with cancer care and treatment is an emerging issue commonly experienced by people affected by cancer, albeit to differing degrees. Individuals are currently the largest non-government contributors to health spending in Australia, providing AUD 30.6 billion during the period 2017–2018 [28]. Australians also spend slightly more than the Organisation for Economic Co-operation and Development (OECD) average on medical expenses [29], with Australia’s health system offering both public and private options for cancer care. While Australia’s health system enables patients to make a personal contribution to their healthcare, peak consumer and cancer control organisations have identified that “bill shock” is prevalent in Australia, and a cause of stress that is difficult to manage [3,30]. High costs can result in Australians in need of healthcare delaying their treatment or forgoing it completely. Financial burden, of which out-of-pocket costs is only one contributing factor, is commonly influenced by multiple circumstances.

### 4.1. Some Population Groups Are at Risk of Greater Out-of-Pocket Costs and Financial Burden

Out-of-pocket costs range significantly. The individual clinical and personal circumstances which influence out-of-pocket costs make it difficult to provide a true average out-of-pocket cost or to compare costs meaningfully. Research suggests that the ongoing costs to the patient of managing side effects of cancer treatment and the illness itself are also significant, having a long-term impact on patients’ quality of life and financial situation [31]. The presence and severity of co-morbidities, such as multiple skin cancers or secondary lymphoedema, can increase out-of-pocket costs and extend the period during which out-of-pocket costs are paid well beyond cancer treatment. As financial burden can be experienced at any time, targeted financial support programs designed and delivered at the time of diagnosis or treatment will miss vital groups of patients at risk of financial burden.

Having private health insurance has consistently been found to be a predictor of higher out-of-pocket costs in Australia, with insured patients paying as much as double compared to the uninsured. Australians can choose to have private cancer care, however, many find their private health insurance coverage is inadequate at the time of treatment [16], and the costs of having treatment in the private sector can influence future treatment decisions [22]. This may challenge a common misconception that private insurance serves as an adequate safety net, and it is ill-advised to assume individuals with private insurance have sufficient financial resources to cover the out-of-pocket costs arising with care in the private sector. Accessibility to care, of which affordability is one component [32], can also influence cancer care in the public sector and it should not be assumed that enrolment in a healthcare subsidy program reduces accessibility barriers. Understanding variations in out-of-pocket costs or service usage can identify where patients are not receiving the care needed. Exploring the reasons why Aboriginal and Torres Strait Islander patients access at least 70% fewer Medicare services compared to non-Indigenous Australians, could direct initiatives which improve access and reduce their increased risk of dying of cancer [10].

Being younger, living in a low-income household and residing in rural and remote areas were supported by other evidence to be strong predictors of increased risk of financial burden [33]. A patient survey found that respondents whose household income was ≤AUD 40,000 were two times more likely to report that out-of-pocket costs had a significant impact on their lives, compared to those earning ≥AUD 130,000 per year [3]. This is likely due to less access to financial resources or support in times of unexpected financial stress. One in eight Australians aged 18 years and over are unable to raise AUD 2000 for an emergency fund within a week [34]. It is critical to identify cancer patients who are at increased risk of financial burden early and support them to access appropriate financial support services. Recognising those at greater risk of financial burden and providing appropriate referral pathways to mitigate this risk is a clear opportunity for clinicians and health services in Australia.

Australians living in areas of socioeconomic disadvantage are 37% more likely to die from cancer than wealthier Australians [2], reinforcing the relationship between vulnerability and financial burden. International studies have also demonstrated the link between disadvantage and poorer health outcomes. The Association of Southeast Asian Nations study found that more than 75% of cancer patients had either died or experienced financial catastrophe within 12 months of diagnosis [35]. Cancer patients in the low-income category of each country had more than five times the odds of experiencing death or financial catastrophe than those with high income [35].

### 4.2. Those Affected by Cancer Will Have Reduced Capacity to Regain Financial Stability

Patients and their families face considerable financial consequences which can affect their household income following a cancer diagnosis. The review findings are supported by supplementary evidence indicating that lifestyle changes, the use of savings, selling of assets, borrowing money, carrying a credit card debt, or increasing credit limits are often implemented when income streams are limited [36]. While a patient’s inability to maintain usual employment, or retain or return to pre-diagnosis employment can exacerbate the experience of financial burden [36], family members and friends who care for cancer patients are also financially impacted by the diagnosis. Analysis conducted by Macmillan Cancer Support in the UK found that nearly one in three carers state their income or household finances are affected by caring due to spending more on travel and other caring-related costs [37]. Cancer patients who need to stop work or decrease hours are unlikely to be financially comfortable [13], pushing many families into poverty [11].

### 4.3. Opportunities to Reduce the Financial Burden and Impact of Cancer Exist

Australia’s health and social systems must improve systemic access to government-funded financial support and assistance services for people affected by cancer. Cancer patients are often unaware of social services available to Australian citizens or permanent residents, [21,22] and similarly, the availability of public health treatment options. Patients with private health insurance often feel obliged to use their insurance or assume that private healthcare is their only option.

Cancer patients express disappointment with the lack of transparency around treatment-related costs and support services [25]. The fear of cost and being unaware of financial support options can lead to cancer patients choosing to delay or forgo recommended treatment. Cancer patients often compromise their standard of living due to increasing costs associated with treatment [36]. While this affects a patient’s quality of life, these coping strategies also impact the health and non-health outcomes of the patient. The strategies are not unique to Australia, [36,38,39] and cause emotional and financial distress to patients and their families, impacting cancer outcomes. Cancer patients need to feel comfortable to discuss their treatment costs with their healthcare professional. Normalising and standardising conversations about costs to allow cancer patients the opportunity to access care without fear or the distress of financial burden and supporting them to choose optimal cancer care without detriment to their financial situation is needed.

### 4.4. Limitations

More than half of the studies were cross-sectional, which limited the ability to identify where and when financial burden occurs, and the cumulative impact it has on individuals and families over time. Investing in the production of longitudinal data would be worthwhile, as well as recognising opportunities where conclusions from population samples can be drawn as most evidence was descriptive. While there is increasing attention towards financial toxicity, few papers have been published which hinders the credibility of the review findings. Identifying the similarities and differences that exist were limited as studies considered different cancer types, age groups, variables, and timelines. Some recall bias may also be present as most studies used self-reported measures to comment on out-of-pocket costs and the influence of financial burden among cancer patients and their families.

## 5. Conclusions

Even with universal health coverage and government-funded social welfare programs, Australians diagnosed with cancer frequently experience financial burden. This review has confirmed the known factors which increase the likelihood of out-of-pocket costs, and situations that influence financial burden, which can help inform where programs and policies need to be directed to meet the needs of financially vulnerable populations. Efforts should be focused on ensuring patients have accurate information on the costs of care and are supported to understand different costs associated with different treatment settings. This information would be invaluable in the context of informed decision making on cancer care and would create greater transparency about out-of-pocket costs between patients and healthcare professionals, and knowledge of low or no cost care options, including the effective use of private health insurance, where available. However, this is only one component of a complex issue to address and therefore, a multi-level approach across individual, health services and the health system levels is necessary.

## Figures and Tables

**Table 2:** Quality assessment of studies.

Criteria	Boyages, 2017 [8]	Callander, 2019 [9]	Callander, 2019 [10]	Dussel, 2011 [11]	Gordon, 2019 [12]	Gordon, 2017 [13]	Gordon, 2017 [14]	Gordon, 2018 [15]	Gordon, 2020 [16]	Hall, 2016 [17]
Was there a clear research question?	Y	Y	Y	Y	Y	Y	Y	Y	Y	Y
Was the study population clearly specified?	Y	Y	Y	Y	Y	Y	Y	Y	Y	Y
Was the participation rate at least 50%?	CD	Y	Y	N	CD	N	NR	Y	NR	N
Were the patients recruited from the same or similar populations? Were inclusion and exclusion criteria applied uniformly?	N	Y	Y	Y	N	Y	Y	N	N	Y
Was there a sample size justification?	Y	Y	Y	Y	Y	Y	NR	Y	Y	Y
Was the exposure(s) prior to the outcome(s) measured?	N	N	N	N	N	N	N	N	N	N
Was the timeframe sufficient?	N	N	N	N	N	CD	N	N	N	N
Were different levels of the exposure as related to the outcome measured?	Y	Y	Y	Y	Y	Y	Y	Y	Y	Y
Were the exposure measures valid and reliable?	N	Y	Y	N	Y	N	Y	Y	Y	N
Was the exposure(s) assessed more than once over time?	N	N	N	N	N	Y	N	N	N	N
Were the outcome measures clearly defined, valid and reliable?	N	Y	Y	N	Y	N	Y	Y	Y	N
Were the outcome assessors blinded to the exposure status of participants?	Y	Y	Y	Y	Y	NR	Y	Y	NR	NR
Was there a loss to follow-up after baseline of 20% or less?	NA	NA	NA	NA	NA	CD	NR	NR	N	NR
Were potential confounding variables measured and adjusted for statistically?	N	Y	Y	N	N	Y	Y	Y	Y	Y
**Criteria**	**Hall, 2015** [18]	**McGrath, 2016** [19]	**McNeil, 2019** [20]	**Newton, 2018** [21]	**Paul, 2016** [22]	**Paul, 2016** [23]	**Rowell, 2016** [24]	**Slavova-Azmanova, 2019** [25]	**Zucca, 2011** [26]	
Was there a clear research question?	Y	CD	Y	Y	Y	Y	Y	Y	Y	
Was the study population clearly specified?	Y	NR	Y	Y	Y	Y	Y	Y	Y	
Was the participation rate at least 50%?	N	NR	NR	N	Y	NR	CD	NR	N	
Were the patients recruited from the same or similar populations? Were inclusion and exclusion criteria applied uniformly?	Y	Y	N	Y	Y	Y	Y	Y	Y	
Was there a sample size justification?	Y	N	Y	N	Y	Y	Y	NR	Y	
Was the exposure(s) prior to the outcome(s) measured?	N	N	N	N	N	N	N	N	Y	
Was the timeframe sufficient?	N	N	N	N	N	N	N	N	Y	
Were different levels of the exposure as related to the outcome measured?	Y	Y	NA	Y	N	NA	Y	N	Y	
Were the exposure measures valid and reliable?	Y	N	N	N	N	N	Y	N	N	
Was the exposure(s) assessed more than once over time?	N	N	N	N	N	N	N	N	Y	
Were the outcome measures clearly defined, valid and reliable?	N	N	N	N	N	N	Y	N	Y	
Were the outcome assessors blinded to the exposure status of participants?	NR	Y	N	N	N	N	NR	N	N	
Was there a loss to follow-up after baseline of 20% or less?	NR	NR	NA	NR	N	NA	NR	N	N	
Were potential confounding variables measured and adjusted for statistically?	Y	Y	Y	Y	Y	Y	Y	NA	Y	

CD, cannot determine; N, no; NA, not applicable; NR, not reported; Y, yes. Green highlight = low risk of bias (good quality).

## Data Availability

No new data were created or analysed in this study. Data sharing is not applicable to this article.

## Supplementary Materials

The following are available online at https://www.mdpi.com/1660-4601/18/5/2422/s1, Table S1: PubMed search strategy (search completed on 1 March 2020).
