# Supplementary material for: Australian Experiences of Out-of-Pocket Costs and Financial Burden Following a Cancer Diagnosis: A Systematic Review
Source: Int J Environ Res Public Health. 2021 Mar 2;18(5):2422. doi: 10.3390/ijerph18052422 (PMC7967550; doi:10.3390/ijerph18052422)
Supplement: Supplementary file 1 [file ijerph-18-02422-s001.pdf]

Table S1. PubMed search strategy (search completed on 1 March 2020).

#	Searches
1	Neoplasm* [MeSH Terms] OR tumo?r* [MeSH Terms] OR tumo?r* [Title/Abstract] OR neoplasm* [Title/Abstract] OR cancer [Title/Abstract] OR malign* [Title/Abstract] OR oncolog* [Title/Abstract] OR carcinoma* [Title/Abstract]
2	Health expenditure* [MeSH Terms] OR high cost* [MeSH Terms] OR out-of-pocket [Title/Abstract] OR financial toxicity [Title/Abstract] OR financial hardship [Title/Abstract] OR financial burden [Title/Abstract]
3	Australia* [MeSH Terms] OR Australia* [Title/Abstract] OR New South Wales [MeSH Terms] OR New South Wales [Title/Abstract] OR Queensland [MeSH Terms] OR Queensland [Title/Abstract] OR Northern Territory [MeSH Terms] OR Northern Territory [Title/Abstract] OR South Australia [MeSH Terms] OR South Australia [Title/Abstract] OR Western Australia [MeSH Terms] OR Western Australia [Title/Abstract] OR Victoria [MeSH Terms] OR Victoria [Title/Abstract] OR Tasmania [MeSH Terms] OR Tasmania [Title/Abstract]
